# Effect of exercise training on psychological outcomes in adults with overweight or obesity: A systematic review and meta‐analysis

**DOI:** 10.1111/obr.13261

**Published:** 2021-05-06

**Authors:** Eliana V. Carraça, Jorge Encantado, Francesca Battista, Kristine Beaulieu, John E. Blundell, Luca Busetto, Marleen van Baak, Dror Dicker, Andrea Ermolao, Nathalie Farpour‐Lambert, Adryan Pramono, Euan Woodward, Alice Bellicha, Jean‐Michel Oppert

**Affiliations:** ^1^ CIDEFES, Universidade Lusófona de Humanidades e Tecnologias, Faculdade de Educação Física e Desporto Lisbon Portugal; ^2^ APPsyCI—Applied Psychology Research Center Capabilities and Inclusion ISPA—University Institute Lisbon Portugal; ^3^ Sport and Exercise Medicine Division, Department of Medicine University of Padova Padua Italy; ^4^ Appetite Control and Energy Balance Group (ACEB), School of Psychology, Faculty of Medicine and Health University of Leeds Leeds UK; ^5^ Obesity Management Task Force (OMTF) European Association for the Study of obesity (EASO); ^6^ Department of Medicine University of Padova Padua Italy; ^7^ NUTRIM School of Nutrition and Translational Research in Metabolism, Department of Human Biology Maastricht University Medical Centre+ Maastricht Netherlands; ^8^ Department of Internal Medicine D, Hasharon Hospital, Rabin Medical Center, Sackler School of Medicine Tel Aviv University Tel Aviv Israel; ^9^ Obesity Prevention and Care Program Contrepoids; Service of Endocrinology, Diabetology, Nutrition and Patient Education, Department of Internal Medicine University Hospitals of Geneva and University of Geneva Geneva Switzerland; ^10^ INSERM, Nutrition and obesities: systemic approaches, NutriOmics Sorbonne University Paris France; ^11^ UFR SESS‐STAPS University Paris‐Est Créteil Créteil France; ^12^ Assistance Publique‐Hôpitaux de Paris (AP‐HP), Pitié‐Salpêtrière hospital, Department of Nutrition, Institute of Cardiometabolism and Nutrition Sorbonne University Paris France

**Keywords:** exercise, obesity, psychological, psychosocial

## Abstract

This study systematically identified the effects of exercise on multiple psychological outcomes among adults with overweight/obesity, also assessing whether these effects differed across exercise types, genders, age, and body mass index (BMI) categories. Pubmed, Web of Science, PsychInfo, and SportDiscus were searched up to October 2019 for peer‐reviewed papers assessing exercise training effects on psychosocial outcomes in adults with overweight/obesity. Thirty‐six articles, 32 randomized controlled trials (RCTs), were included in this review. Most interventions were supervised (65%), ranging between 6 and 76 weeks (median = 12). Sixteen psychological outcomes were studied. Exercise induced positive changes in quality of life but did not reduce depression. Large effect sizes were observed on quality of life's physical component, but exercise was also able to improve vitality and mental health. Most psychological outcomes (e.g., body image, anxiety, and perceived stress) are poorly studied, evidencing either conflicting or null exercise effects. Exercise self‐efficacy and autonomous motivations were also consistently improved. Exercise types and gender seem to moderate exercise psychological effects. Exercise training programs might lead to positive changes in some psychological outcomes, especially in quality of life, in adults with overweight and obesity, but more studies, with greater systematization in program characteristics, and longer follow‐ups are still required to allow more solid conclusions.

## 1 INTRODUCTION

In the midst of the modern epidemic of obesity there is an urgent call for lifestyle changes.^1^ These changes are large in scope, from environmental and political to individual changes in self‐regulated behaviors such as physical (in)activity and (un)healthy eating.^2^ Indeed, the choices we make every day may have a significant impact on the prevention (or not) of life‐long detrimental chronic diseases,^3^ definitely the most prevalent nowadays in people with excess weight or obesity.^4^, ^5^ Furthermore, obesity has a pronounced negative impact on the psychological health of individuals. Studies have found associations between obesity and mental disorders, such as anxiety or depressive disorders.^6^, ^7^ Increased incidence of body dissatisfaction,^8^ poor quality of life,^9^ depression, anxiety and somatoform disorders^10^ was also observed among individuals with overweight and obesity.

Physical activity is one of the cornerstones in the reduction of obesity and overweight rates, as it supports weight management^11^ and is associated with substantial health benefits, lower risks of all‐cause cardiovascular disease and cancer‐related mortality.^12^, ^13^ Exercise/physical activity may also attenuate the negative psychological outcomes associated with obesity. Various meta‐analyses have suggested that exercise is linked to lower levels of depression^14^ and anxiety,^15^ improved quality of life,^16^ and more positive body image^17^ in the general population.

However, in populations with overweight and obesity, these benefits are not so clear. In a recent systematic review evaluating exercise‐induced effects on four psychological outcomes in adults with obesity (body mass index ([BMI] > 29 kg/m^2^), Baillot et al.^18^ did not find significant exercise effects on quality of life and depression outcomes. Studies examining exercise effects on body image and anxiety were too few to allow quantitative analysis. Another systematic review including postmenopausal women with overweight and obesity reported contradicting findings: apparently no exercise effects on anxiety, depression and stress, and either positive or non‐significant effects of physical activity on quality of life.^19^ When looking only into the effects of resistance exercise, a recent systematic review with populations with overweight and obesity of all ages found weak positive effects on some psychological outcomes (self‐efficacy, self‐esteem, inhibition, and psychological disorders like anxiety) but the data precluded solid conclusions.^20^

Although relatively recent, prior reviews have either failed to include adults with overweight,^18^ included only one gender,^19^ included mixed child, adolescent and adult samples,^20^ included a behavioral component which prevents a real appraisal of exercise effects,^20^ only evaluated acute exercise effects,^21^ or left out important psychological outcomes like self‐efficacy and motivations.^18^ Providing that psychological health benefits of exercise have been proposed to mediate successful adherence to weight‐related behaviors and subsequent weight loss,^22^, ^23^, ^24^, ^25^ there is a need to clarify the psychological effects of exercise and physical activity among individuals with excess weight and obesity.

Building upon the efforts of the EASO Physical Activity Working Group, the current systematic review and meta‐analysis investigated the effects of supervised or semi‐supervised physical activity and/or exercise on multiple psychological outcomes among adults with overweight or obesity and to assess whether the effects differ across types of exercise (e.g., aerobic, resistance), gender, age and BMI category.

## 2 METHODS

This systematic review follows the Preferred Reporting Items for Systematic Reviews and Meta‐Analysis (PRISMA) guidelines^26^ and is registered in the PROSPERO database (registration number CRD42019157823).

### 2.1 Search strategy

Four electronic databases (PubMed, Web of Science, PsychInfo and SportDiscus) were searched for original articles published in English up to October 2019 (including online ahead of print publication). A comprehensive search strategy, combining terms concerning: (i) the population of interest (obesity; adults); (ii) the evaluated exposure(s) (e.g., exercise, physical activity, resistance training); (iii) the outcomes of this review (e.g., quality of life, body image, self‐efficacy, depression); and (iv) the study design (e.g., randomized controlled trial or controlled clinical trial or intervention or program [me] or experimental). A full search example can be found in Table S1. Previous systematic reviews were screened to identify relevant subject headings and keywords to include within each subject category. Reference lists from the resulting reviews and articles were also screened to identify additional articles.

### 2.2 Study selection, inclusion, and exclusion

Articles were included if they involved adults (≥18 years) with overweight (BMI ≥ 25 kg/m^2^) or obesity (BMI ≥ 30 kg/m^2^) participating in supervised or semi‐supervised exercise interventions, and assessed one or more psychosocial outcomes (both pre‐exercise and post‐exercise or compared with control). Randomized controlled trials (RCT), non‐randomized controlled trials, quasi‐experimental studies, or single‐group intervention studies were eligible study designs. Studies involving multicomponent interventions (e.g., exercise paired with a behavioral intervention or diet) were excluded if the isolated effect of exercise could not be determined (ex. diet + exercise vs. control; behavioral intervention vs. control). Studies focusing on the primary prevention of weight gain/obesity were not included. Presence of obesity comorbidities, namely type 2 diabetes, hypertension, dyslipidaemia, metabolic syndrome, liver disease (NAFLD/NASH), and osteoarthritis, was not an exclusion criterion (rf summary paper for details). No minimum intervention length criterion was applied.

Abstracts and full texts were assessed for eligibility independently by two authors (EVC, JE) with uncertainty regarding eligibility discussed among authors.

### 2.3 Data extraction and synthesis

Data was extracted by two authors using standardized forms. The following characteristics were retrieved from each article: reference, study design, number of participants included in intervention and control groups, population characteristics (age, BMI, % female, comorbidities for intervention and control groups), trial characteristics (program description and comparison, length, and follow‐up), psychological outcomes and instruments, and main results.

### 2.4 Quality assessment

To assess study quality we used the tool developed by the National Heart, Lung and Blood Institute (NHLBI, USA) that has been previously used for defining guidelines for the management of obesity.^27^ The original assessment forms for controlled/comparative trials and single‐group intervention studies were used. These forms comprise several items, answered on a yes/no basis, tapping numerous aspects deemed to affect the risk of bias like study design, dropout, and compliance rates, eligibility criteria, data collection, power analysis, and intent‐to‐treat analysis. In the first form, four of these items represented fatal flaws if answered “No/Not reported/Can't determine,” namely (i) randomization, (ii) dropout rate <20%, (iii) valid/reliable outcome measures, (iv) intent‐to‐treat analysis. In the second form, three items were considered fatal flaws if not met, namely, (i) eligibility criteria pre‐defined, (ii) sufficient sample size, and (iii) dropout rate <20% or intent‐to‐treat analysis. A global rating was determined based on the number of fatal flaws: good quality (0 fatal flaws), fair quality (1 fatal flaw), or poor quality (≥2 fatal flaws). Quality assessment was conducted independently by two reviewers (EVC, JE), and disagreement was resolved through discussion (with a third author where necessary).

### 2.5 Data analysis

A narrative synthesis of results, organized by psychological outcome and study design, was performed. Random‐effect meta‐analyses, using controlled trials with non‐active controls, were conducted using the Comprehensive Meta‐Analysis (CMA) Software version 3.3.070.^28^ Random effects models were chosen due to the considerable heterogeneity expected among studies.

Effect sizes were computed based on post‐intervention scores for intervention and control groups, using means (M), standard deviations (SD) and sample sizes for each group, or based on the mean difference (and SD of the difference). When these data were missing, effect sizes were obtained from alternative parameters (e.g., means and standard errors or interquartile ranges). Effect sizes were interpreted according to Cohen's^29^ guidelines (values of 0.20, 0.50, and 0.80 for small, medium and large effect sizes, respectively). The 95% CI, Z values and corresponding *p* values were considered as indicators of the significance of the effect. We also inspected the standard residuals for outliers (>1.96).

Heterogeneity was tested using the *I* ^2^ statistic^30^ and the Cochran's *Q* statistic.^31^ The *I* ^2^ ranges from 0 to 100%, where a value of 0% indicates no observed heterogeneity and values of 25%, 50% and 75% reflect low, moderate, and high heterogeneity, respectively.^30^ The Cochran's *Q* statistic demonstrates that studies do not share a common effect size (i.e., there is heterogeneity) when a significant *p* value (<0.05) is found.^31^

The potential for publication bias was subjectively assessed by inspecting funnel plots for asymmetry. They were quantitatively assessed using Egger's test^32^ and Duval and Tweedie's trim‐and‐fill method^33^ when 10 or more studies were available per meta‐analysis and no substantial heterogeneity was present, because the power is too low to distinguish chance from real asymmetry.^34^

To explore heterogeneity within main effects analyses, subgroup analyses were used to assess the impact of type of exercise, gender, age and BMI category.

Sensitivity analyses were carried out to explore the impact of risk of bias on effect sizes by repeating primary analyses with the exclusion of studies/arms with: (1) poor quality and (2) detected as outliers (>1.96).

## 3 RESULTS

**FIGURE 1:**
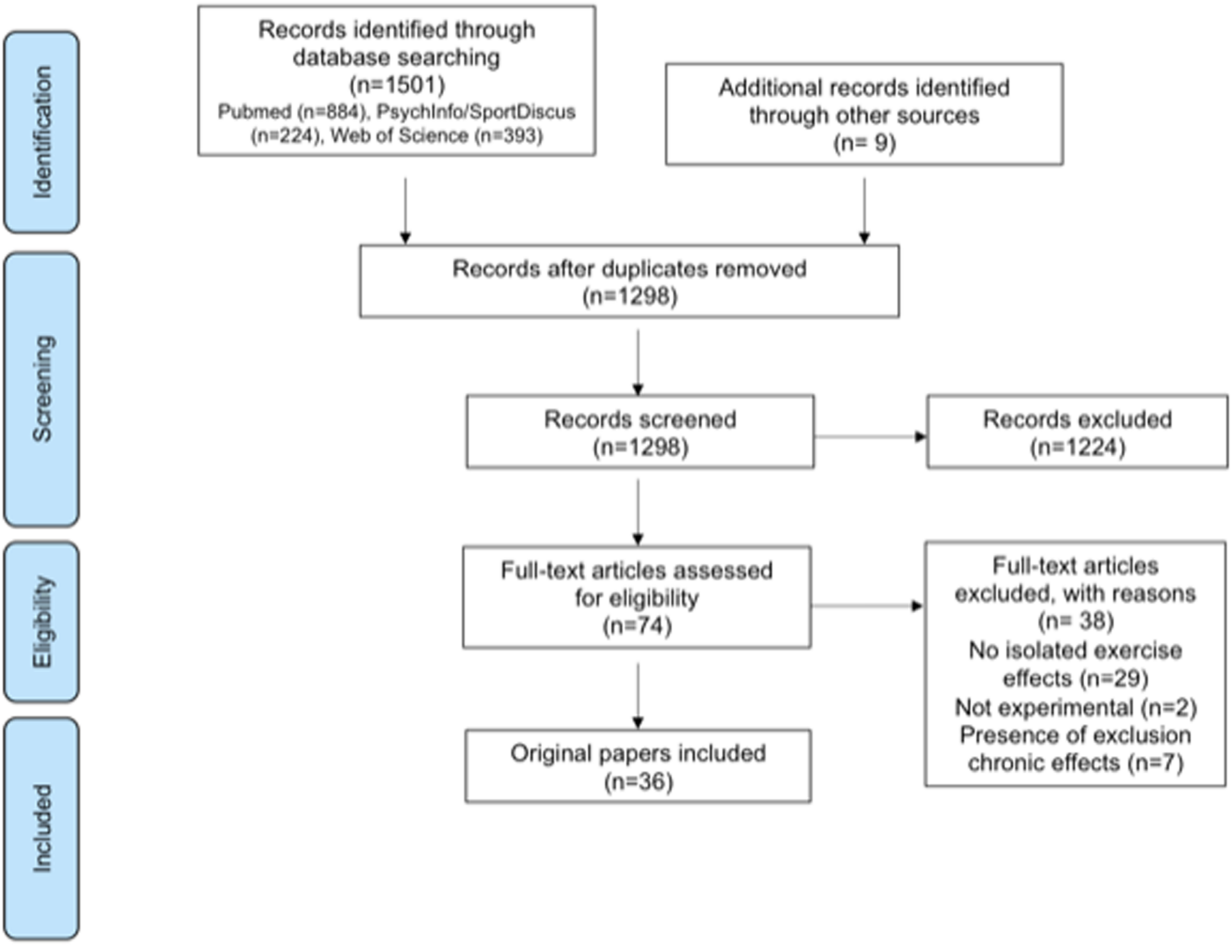
Systematic review flow diagram

Figure 1 illustrates the systematic review flow diagram. The database search yielded 1298 articles after duplicates were removed, 1224 of which were eliminated based on titles and abstracts alone. The full text was retrieved from 74 articles. Thirty‐six articles, corresponding to 31 original studies, satisfied the inclusion criteria and were included in the present systematic review. Twenty‐one studies were included in meta‐analysis.

### 3.1 Study characteristics

The characteristics of the included studies are presented in Table 1. Studies were published between 2000 and 2019. Studies included randomized (n = 32) clinical controlled (n = 1) or non‐controlled (n = 3) trials. These 36 studies represent 3536 participants. The number of participants in each study ranged from 8 to 561 (median, 72 participants). The mean age of trial participants ranged from 32 to 76 years (median, 55 years). The majority of participants were female (74% average). Mean BMI ranged from 27 to 44 kg/m^2^ (median, 31 kg/m^2^). Eleven studies recruited women only and one trial included men only. Eight original studies included subjects with comorbidities: metabolic syndrome^35^ or diabetes.^36^, ^37^, ^38^, ^39^, ^40^, ^41^, ^42^

**TABLE 1:** Characteristics of included original studies

Reference study design	Intervention arms	N by arm (% female)	Age (years)	BMI (kg/m^2^)	Exercise program	Length	Psychological outcomes (instruments)	Main results
Ballin 2019 RCT	Exercise	36 (52)	70 ± 0.2	29 ± 3	Supervised interval training, 3 × 33–51 min/week (progressive), at vigorous intensity (RPE 7 of 10).	10	Health‐related QOL (SF‐36)	The exercise group improved QOL mental component and mental health compared to controls.
	Control	36 (52)						
Batrakoulis 2019 RCT	Exercise (TR)	14 (100)	36 ± 4	29 ± 3	Supervised aerobic and resistance circuit training, 3×/week, 8–12 stations, progressive volume and intensity	40	Psychological health (GHQ‐12) Subjective vitality (SVS) Exercise regulations (BREQ)	Psychological health and vitality was higher post‐intervention in both exercise groups compared to controls. TR showed lower external regulation and higher intrinsic and identified regulations for exercise post‐intervention.
	Exercise + detraining	14 (100)	36 ± 4	28 ± 3				
	Control	21 (100)	36 ± 4	29 ± 3				
Cheema 2015 RCT	Boxing	6 (50)	43 ± 19	32 ± 6	Supervised boxing: 4 × 50 min/week, 5 interval‐based exercises on a 2:1 ratio (>75% HR max). Unsupervised brisk walking: 4 × 50 min/week.	12	Health‐related QOL (SF‐36)	The boxing group improved physical functioning, general health, and vitality, while the walking group decreased vitality (this one also different between groups).
	Walking (control)	6 (67)	36 ± 15	31 ± 3				
Domene 2015 RCT	Exercise	10 (100)	33 ± 11	27 ± 2	Supervised Zumba classes, 1–2 × 60 min/week	8	Health‐related QOL (SF‐36)	Improvements in physical functioning, general health, energy/fatigue and emotional wellbeing were observed in the exercise group, and no changes in the controls.
	Control	10 (100)	35 ± 13	28 ± 2				
Fang 2018 RCT	Exercise	37 (46)	37 (range 22–50)	NR	Semi‐supervised aerobic training, 3 × 60 min/week, moderate intensity	12	Health‐related QOL (WHOQOL‐BREF) Occupational job stress (OWSI) Job satisfaction (MSQ)	Significant differences in all QOL domains, job satisfaction and 2 dimensions of work stress (work control and interpersonal relationship) favoring the exercise group were observed.
	Control	38 (34)						
Focht 2005 RCT Adapt *Osteoarthritis	Diet	82 (72)	69 ± 0	35 ± 1	Semi‐supervised aerobic (50–75% HR res) and resistance training (2 sets, 12 reps, 4 exercises), 3 × 60 min/week	76	Walking self‐efficacy (WSES) Stair‐climbing self‐efficacy (NR) Pain (WOMAC)	The diet + exercise group showed significant improvements in walking and stair‐climbing self‐efficacy and in pain vs. controls. The exercise group also improved walking self‐efficacy vs. controls.
	Exercise	80 (74)	68 ± 1	34 ± 1				
	Diet + Exercise	76 (74)	69 ± 1	34 ± 1				
	Control	78 (68)	69 ± 1	34 ± 1				
Fritz 2011 RCT	Exercise	87 (56)	60 (range 56–64)	29 (range 27–34)	Semi‐supervised Nordic walking, increase 5 h each week for 4 months	16	Health‐related QOL (SWED‐QUAL)	Sleep quality improved with exercise in the normal glucose tolerance and Type‐2‐diabtes subgroups. General health perceptions also improved in the first subgroup.
	Control	115 (60)	61 (range 55–64)	29 (range 27–38)				
Grant 2004 RCT	Exercise	13 (100)	55 ± 7	30 ± 5	Supervised multicomponent program 2 × 40 min/week involving aerobic (20 min), resistance (6 exercises, 5 min), and flexibility (5 min) training	12	Life satisfaction (LSQ) Physical self‐perceptions (PSPP)	Exercisers significantly improved life satisfaction compared to controls. Physical self‐perceptions did not differ from controls. Body attractiveness improved in both groups.
	Control	13 (100)	55 ± 7	30 ± 5				
Heiestad 2016 RCT	Body Pump	35 (100)	38 ± 11	30 ± 5	Full‐body resistance training 3 × 45–60 min/week. Body pump: 30–1222 reps, no rest, and participants selected loads. Other 2 groups performed 1–4 sets, 3–15 reps (range 60–90% 1RM), pauses 30″‐2′	12	General health (SF‐36) Life satisfaction (SWLS) Exercise regulations (BREQ)	All 3 exercise groups showed improvements in RAI, identified and integrated regulations for exercise. General health and life satisfaction did not change.
	Personal training	32 (100)	38 ± 9	32 ± 6				
	Unsupervised exercise	30 (100)	41 ± 11	31 ± 5				
	Control	32 (100)	42 ± 10	31 ± 5				
Herring 2014 RCT	Aerobic + WLT	10 (70)	NR	44 ± 4	Semi‐supervised aerobic training: 3 × 60 min/week, 50–70% HR res. Semi‐supervise resistance training: 3 × 60 min/week, compound exercises, at 60% 1 RM.	12	Anxiety and depression (Hospital Scale) Exercise self‐efficacy (SERPA) Motives for PA (NR)	Self‐efficacy and interest/enjoyment for exercise increased in both exercise groups vs. controls. Competence (i.e., skill development) increased only in the resistance group.
	Resistance + WLT	10 (80)		42 ± 5				
	WLT (control)	7 (57)		49 ± 8				
Imayama 2011a RCT	Exercise	77 (43)	55 ± 7	30 ± 5	Semi‐supervised moderate‐to‐vigorous aerobic exercise 6 × 60 min/week (60–85% HR max).	52	Health‐related QOL (SF‐36) Exercise self‐efficacy (ESES)	Higher vitality score in overweight male exercisers vs. controls. Exercise self‐efficacy was significantly higher in exercisers vs. controls.
	Control	82 (45)	55 ± 7	30 ± 5				
Imayama 2011b RCT	Diet	118 (100)	58 ± 6	31 ± 4	Semi‐supervised moderate‐to‐vigorous aerobic exercise 5 × 45 min/week (70–85% HR max at week 8)	52	Health‐related QOL (SF‐36) Depression and anxiety (BSI‐18) Perceived stress (PSS)	No significant differences between exercise and control groups, but physical functioning, role‐physical and vitality were higher in the combined (vs. diet) group.
	Exercise	117 (100)	58 ± 5	31 ± 4				
	Diet + Exercise	117 (100)	58 ± 5	31 ± 4				
	Control	87 (100)	57 ± 4	31 ± 4				
Levinger 2007 RCT *Metabolic syndrome risk factors	HiMF + StEx	15 (33)	52 ± 7	32 ± 4	Supervised resistance training 3 × 60 min/week, 8 exercises, 2–3 sets (progressing from 15–20 rep at 40–50% 1RM to 8–12 rep at 75–85% 1RM)	10	Health‐related QOL (SF‐36)	Strength exercise increased physical and mental health (12.9 and 8.3%, respectively) in the HiMF + StEx vs. HiMF + control
	LoMF + StEx	12 (33)	51 ± 5	24 ± 3				
	HiMF + control	15 (33)	52 ± 6	30 ± 4				
	LoMF + control	13 (31)	49 ± 8	24 ± 3				
Levinger 2011 RCT *Metabolic syndrome risk factors	HiMF + StEx	15 (33)	52 ± 7	32 ± 4	Supervised resistance training 3 × 60 min/week, 8 exercises, 2–3 sets (progressing from 15–20 rep at 40–50% 1RM to 8–12 rep at 75–85% 1RM)	10	Depression (CDS)	Strength exercise decreased (improved) depressed mood compared to baseline and to the HiMF + control
	LoMF + StEx	12 (33)	51 ± 5	24 ± 3				
	HiMF + control	15 (33)	52 ± 6	30 ± 4				
	LoMF + control	13 (31)	49 ± 8	24 ± 3				
Lim 2010 RCT	Aquatic exercise	26 (88)	66 ± 9	28 ± 2	Supervised aquatic exercise, 3 × 40 min/week, >65% HR max. Supervised land‐based exercise, 3 × 40 min/week, joint mobilization and strength exercises at 40–60% 1 RM.	8	Health‐related QOL (SF‐36) Pain (BPI)	The aquatic group showed significant improvements in pain interference vs. controls. Within changes in quality of life were observed in both exercise groups.
	Land‐based exercise	25 (84)	68 ± 8	28 ± 2				
	Control	24 (88)	63 ± 5	28 ± 2				
Martins 2011 RCT	Aerobic	24 (67)	76 ± 7	29 ± 4	Supervised aerobic training, walking 3 × 45 min/week, (progressive intensity, 40–85% HR reserve). Supervised resistance training: 8 calisthenics and elastic bands exercises, 3×/week, 1–3 sets, 8–15 reps (moderate intensity).	16	Mood states (POMS)	No differences between groups. Significant within‐increases in vigor in the resistance group.
	Resistance	23 (57)	73 ± 6	29 ± 4				
	Control	31 (58)	78 ± 9	30 ± 5				
Megakli 2016 RCT	Exercise	35 (100)	32 ± 7	36 ± 5	Supervised aerobic and strength training 3 × 30–40 min/week	12	Health‐related QOL (SF‐36) Exercise self‐efficacy (NR)	Exercise increased exercise self‐efficacy, physical functioning, vitality, bodily pain, mental health, and role‐emotional, but not social functioning, general health, or role‐physical.
	Control	37 (100)	32 ± 8	37 ± 5				
Megakli 2017 RCT	Exercise	35 (100)	32 ± 7	36 ± 5	Supervised aerobic and strength training 3 × 30–40 min/week	12	Self‐esteem (RSES) Physical self‐perceptions (PSPP)	Exercise increased physical self‐worth and self‐esteem, but not body attractiveness.
	Control	37 (100)	32 ± 8	37 ± 5				
Messier 2013 RCT Idea *Osteoarthritis	Diet + Exercise	152 (72)	65 ± 6	34 ± 4	Semi‐supervised aerobic and resistance training, 3 × 60 min/week	76	Health‐related QOL (SF‐36) Pain (WOMAC)	Diet + Exercise group showed greater scores in QOL physical component and less pain post‐intervention than the diet group.
	Diet	152 (71)	66 ± 6	34 ± 4				
	Exercise	150 (72)	66 ± 6	34 ± 4				
Messier 2010 RCT	Diet + Exercise	36 (100)	57 ± 5	33 ± 5	Supervised resistance training: 3×/week, 2–4 sets, 8–15 reps, 6 exercises (organized on 4 progressive phases).	24	Health‐related QOL (MOSGHS) Perceived stress (PSS) Self‐esteem (RSES) Body esteem (BES) Weight self‐efficacy (NR)	The addition of resistance training to caloric restriction had no additional benefit on the psychosocial profile. Both groups improved weight self‐efficacy, body esteem, self‐esteem, and health perceptions (QOL).
	Diet	71 (100)	58 ± 5	32 ± 5				
Mihalko 2019 RCT Idea *Osteoarthritis	Diet + Exercise	152 (72)	65 ± 6	34 ± 4	Semi‐supervised aerobic and resistance training, 3 × 60 min/week	76	Balance activities self‐efficacy (ABCS) Walking self‐efficacy (WES) Gait efficacy (GSES)	Diet + Exercise group showed greater scores for all three self‐efficacy measures post‐intervention than the diet group.
	Diet	152 (71)	66 ± 6	34 ± 4				
	Exercise	150 (72)	66 ± 6	34 ± 4				
Napoli 2014 RCT	Diet	26 (65)	70 ± 4	37 ± 5	Supervised multicomponent program 3 × 90 min/week involving aerobic (30 min, 65–85% HR peak), resistance (8 exercises, 1–3 sets, 6–12 rep at 65–80% 1RM), and balance (15 min) and flexibility (15 min) training	52	Obesity‐specific QOL (IWQOL) Depression (GDS)	There were significant differences in all variables but self‐esteem between exercise vs. control, and between diet + exercise vs. diet
	Exercise	26 (61)	70 ± 4	37 ± 5				
	Diet + Exercise	28 (57)	70 ± 4	37 ± 5				
	Control	27 (67)	69 ± 4	37 ± 5				
Nieman 2000 RCT	Diet	26 (100)	46 ± 1	33 ± 1	Semi‐supervised walking 5 × 45 min/week (60–80% HR max)	12	Psychological wellbeing (GWBS) Mood states (POMS)	Exercise alone did not lead to significant changes in psychological wellbeing and mood states compared to controls.
	Exercise	21 (100)	(overall)	overall				
	Diet + Exercise	22 (100)						
	Control	22 (100)						
Nishijima 2007 RCT *Hypertension, hyperlipidemia or glusoce intolerance	Exercise	281 (59)	67 ± 7	27 ± 2	Semi‐supervised aerobic exercise on cycle‐ergometer, at 40–70% VO_2_ Peak, complemented with 4 resistance exercises in the later stages (2 sets, 20 reps), 60–90 session	24	Health‐related QOL (SF‐36)	General health, vitality and mental health improved significantly more in the exercise group than in the control group.
	Control	280 (58)	67 ± 7	27 ± 2				
Plotnikoff 2010 RCT *Type 2 diabetes	Exercise	27 (70)	55 ± 12	35 ± 8	Semi‐supervised strength training 3×/week, 2–3 sets, 8–12 reps for 8 exercises (50–80% 1‐RM)	16	Health‐related QOL (SF‐12) Task, scheduling and barrier self‐efficacy measures (Plotnikoff et al. scales)	There were no significant differences between the exercise and control groups in health‐related quality of life. Scheduling self‐efficacy (confidence to do regular exercise) was significantly higher in the exercise group vs. controls.
	Control	21 (72)	54 ± 12	36 ± 5				
Rejeski 2002 RCT Adapt *Osteoarthritis	Diet	82 (72)	69 ± 0	35 ± 1	Semi‐supervised aerobic (50–75% HR res) and resistance training (2 sets, 12 reps, 4 exercises), 3 × 60 min/week	76	Health‐related QOL (SF‐36) Body satisfaction (Reboussin et al.)	The exercise group showed significantly higher satisfaction with appearance and body function than controls. The diet + exercise group showed higher satisfaction with body function than the diet group.
	Exercise	80 (74)	68 ± 1	34 ± 1				
	Diet + Exercise	76 (74)	69 ± 1	34 ± 1				
	Control	78 (68)	69 ± 1	34 ± 1				
Sarsan 2006 RCT	Aerobic	26 (100)	42 ± 8	35 ± 5	Supervised aerobic training: walking followed by cycle‐ergometer (progressive) 3–5×/week 12–45 min (50–85% HR reserve). Supervised resistance training: 3×/week, 1–3 sets, 10 reps, 6 exercises (40–80% 1‐RM).	12	Depression (BDI)	Depression significantly decreased in the aerobic group compared with controls, but it was not significantly different from controls in the resistance group. Still, both exercise groups improved depression scores from baseline.
	Resistance	26 (100)	43 ± 10	34 ± 3				
	Control	24 (100)	44 ± 7	36 ± 4				
Sukala 2013 RCT *Type 2 diabetes	Aerobic	13 (54)	51 ± 4	45 ± 7	Supervised aerobic training: cycle‐ergometer, 3×/week, 40–60 min (65–85% HR reserve). Supervised resistance training: 3×/week, 2–3 sets, 6–8 reps for 8 exercises (progressively adjusted weight)	16	Health‐related QOL (SF‐36)	No significant differences between groups. Improvements in quality of life occurred in both groups, especially in the physical component.
	Resistance	13 (46)	48 ± 6	43 ± 12				
Svensson 2017 RCT	High intensity	49 (76)	44 ± 8	42 ± 5	Aerobic plus resistance training. High intensity: supervised, 3 × 60 min/week (85–95% HR max). Moderate intensity: semi‐supervised, 3 × 30 min (76–85% HR max) and 3 × 30 min (40–55% HR max)/week.	16	Health‐related QOL (SF‐36)	There were significant differences in QOL physical component between the high intensity group and controls, in particular in physical function and general health. Within‐changes were observed in QOL mental component in the high intensity group.
	Moderate intensity	39 (74)	47 ± 10	43 ± 8				
	Control	22 (73)	47 ± 9	45 ± 7				
Tamin 2018 RCT *Osteoarthritis	Aquatic exercise	15 (NR)	61 ± 43	NR	Supervised aquatic exercise: combination of aerobic and resistance exercises. Land‐based exercise: cycle‐ergometer 50 rpm, 15–45 min (progressive).	8	Health‐related QOL (SF‐36)	There were improvements in QOL in both groups, but no significant between‐group differences. Energy/fatigue and emotional wellbeing, and general health improved in the aquatic group. Land‐based improved energy/fatigue, role‐physical, role‐emotional, and pain.
	Land‐based exercise	18 (NR)	65 ± 49					
Vancini 2017 RCT	Pilates	22 (95)	56 ± 7	31 ± 4	Supervised Pilates: 3 × 45 min mat exercises, using reformers, Cadillacs, chairs, magic circles, and dumbbells. Supervised walking: 3 × 60 min/week, moderate aerobic training in flat terrain.	8	Health‐related QOL (SF‐36) Depression (BDI) Anxiety (STAI)	The walking group improved mental health and state anxiety vs. controls. The Pilates group improved mental health vs. controls. Both exercise groups improved from baseline in some QOL domains, depression, and anxiety.
	Walking	21 (95)	42 ± 7	30 ± 3				
	Control	20 (60)	42 ± 13	32 ± 4				
Villareal 2011 RCT (Same study as Napoli 2014)	Diet	26 (65)	70 ± 4	37 ± 5	Supervised multicomponent program 3 × 90 min/week involving aerobic (30 min, 65–85% HR peak), resistance (8 exercises, 1–3 sets, 6–12 rep at 65–80% 1RM), and balance (15 min) and flexibility (15 min) training	52	Health‐related QOL (SF‐36)	There were significant differences in QOL physical component between the exercise and control groups, but none in the mental component. No other differences were found between groups.
	Exercise	26 (62)	70 ± 4	37 ± 5				
	Diet + Exercise	28 (57)	70 ± 4	37 ± 5				
	Control	27 (67)	69 ± 4	37 ± 5				
Rica 2013	Exercise	28 (100)	69 ± 6	33 ± 4	Supervised water exercise, 3 × 60 min/week (70% age‐predicted HR max), 10–15 reps of each exercise	12	Health‐related QOL (WHO questionnaire)	There were significant differences in QOL physical, psychological, social and environmental domains between the exercise and control groups.
NRCT	Control	10 (100)	68 ± 4	34 ± 3				
Baillot 2012 SGIS * Type 2 diabetes	Exercise	8 (0)	60 ± 2	36 ± 2	Supervised walking (2 × 45 min/week, 75% HR peak) and cycle‐ergometer (1 × 45 min/week, intermittent exercise, 2 min at 85% HR peak + 3 min at 60% HR peak)	8	Obesity‐specific QOL (QOLOD)	QOL psychosocial domain significantly increased from pre to post‐intervention.
Cugusi 2018 SGIS	Exercise	18 (100)	38 ± 11	28 ± 2	Supervised mini‐trampoline rebound exercise, 3 × 55–60 min, 40–90% HR res	12	Health‐related QOL (SF‐36) Pain (BPI‐SF)	Physical functioning, vitality, social functioning, role‐emotional, and the mental component summary improved, as well as pain scores.
Wouters 2010 SGIS	Exercise	15 (87)	44 (range 28–60)	38 ± 5	Supervised aquajogging, 2 × 60 min/week (stimulated to gradually increase the intensity)	6	Obesity‐specific QOL (IWQOL) Exercise self‐efficacy (ESES)	Physical function, self‐esteem, and public distress significantly improved from pre to post‐intervention.

Abbreviations: NR, not reported; NRCT, non‐randomized controlled trial; QOL, quality of life; RCT, randomized controlled trial; SGIS, single‐group intervention study.

Interventions delivered ranged from 6 to 76 weeks (median, 12 weeks) and most were supervised (65%). Thirteen studies involved an aerobic‐only exercise program, eight studies comprised a resistance‐only exercise program, and six studies involved a combined aerobic plus resistance exercise program. A few studies included water exercise,^42^, ^43^, ^44^, ^45^ pilates,^46^ and multicomponent programs also involving balance or flexibility exercises.^47^, ^48^ Twenty‐four studies involved a passive control, two studies combined exercise with diet and involved a diet‐only control, and two studies were comparative, involving two different exercise groups. Exercise session duration ranged from 12 to 90 min (median, 60 min), with a frequency between one and six sessions per week (median, three sessions), and intensity generally varying between moderate and vigorous. Eleven studies did not report exercise intensity, four did not report session duration and three did not report the exact number of planned sessions per week.

Several psychological outcomes were measured in the included studies. Twenty‐five studies examined exercise effects on health‐related hquality of life, six studies measured exercise effects on depression, four studies examined exercise‐induced effects on body image and perceived pain, three studies explored exercise effects on anxiety and obesity‐specific quality of life, two studies tested exercise effects on life satisfaction, perceived stress, self‐esteem, and mood states. Exercise effects on a few context‐specific psychological measures were also examined, namely on exercise motivations (n = 2), self‐efficacy measures (e.g., for exercise, weight, gait, stair‐climbing; n = 8 overall), motives for physical activity (n = 1), job‐related stress (n = 1), and job satisfaction (n = 1).

### 3.2 Study quality

Of the 36 studies identified as relevant for this review, the methodological quality of 14 studies was rated as good, 14 were classified as fair, and eight were rated as poor. Regarding the study main flaws, of the 36 studies, four did not use randomized controlled designs, 11 presented dropout rates above 20%, 16 did not perform intent‐to‐treat analysis, and three did not have sufficient sample size according to initially estimated sample size requirements (see Table S2).

### 3.3 Exercise‐induced effects on quality of life

Twenty‐five studies (23 RCT, 1 non‐randomized controlled trial, and 1 single‐group intervention study) measured at least one subcomponent/component of health‐related quality of life using general measures. Of these, 18 studies (72%) used SF‐36, one used SF‐12, two used the WHO Quality of Life Questionnaire, two used adapted versions of the SF‐36, and two used questionnaires to assess specific subcomponents of quality of life (general health and vitality). Three additional studies (one RCT and two single‐group intervention studies) employed obesity‐specific quality of life measures, of which 2 used the Impact of Weight on Quality of Life Questionnaire. Twenty of the 25 studies (80%) using general measures reported positive effects of exercise on at least one subcomponent/component of health‐related quality of life, especially on vitality (69%), mental health (67%), and physical component (60%). Regarding obesity‐specific quality of life, two of three studies showed exercise‐induced improvements on physical functioning and public distress.^44^, ^49^

Meta‐analyses were conducted for each quality of life component and subcomponent derived from SF‐36 (Table 2). The number of studies included ranged from 8 to 11 (10 to 14 arms) and the number of participants ranged from 492 to 960 participants (303–561 from intervention groups and 189–399 from controls). Exercise had significant positive effects, representing a large effect size, on quality of life overall physical component (SMD 0.90, 95% CI 0.29–1.51). Small magnitude significant effect sizes were found for three of the four physical subcomponents, namely physical functioning (0.40, 95% CI 0.14–0.66), role‐physical (0.31, 95% CI 0.03–0.58), and bodily pain (0.24, 95% CI 0.08–0.40). Effects were not significant for the overall mental component, but small magnitude significant effect sizes were found for vitality (0.41, 95% CI 0.15–0.68) and mental health (0.22, 95% CI 0.08–0.37) subcomponents.

**TABLE 2:** Meta‐analyses results for exercise effects on quality of life and depression

Study characteristics	Meta‐analyses					
	Sample size IG/CG	SMD	95% CI	Z	*Q*	*I* ^2^
*Quality of Life*						
Physical component (n = 10)	303/189	0.90	0.29, 1.51	2.90**	77.5***	88.4
Mental component (n = 12)	331/210	0.30	−0.30, 0.90	0.99	104.4***	89.5
Physical functioning (n = 11)	492/398	0.40	0.14, 0.66	3.02**	27.4**	63.5
Role‐physical (n = 10)	486/372	0.31	0.03, 0.58	2.20*	29.0**	69.0
Bodily pain (n = 10)	486/372	0.24	0.08, 0.40	2.94**	10.8	17.0
General health (n = 14)	561/399	0.14	−0.02, 0.29	1.71§	15.6	16.5
Vitality (n = 13)	520/399	0.41	0.15, 0.68	3.03**	36.5***	67.1
Social functioning (n = 10)	486/372	0.28	−0.01, 0.57	1.91§	32.2***	72.0
Role‐emotional (n = 10)	486/372	0.16	−0.02, 0.37	1.67§	15.1	40.5
Mental health (n = 10)	486/372	0.22	0.08, 0.37	3.01**	9.40	4.21
*Depression* (n = 8)	273/205	−0.13	−0.53, 0.26	−0.66	26.3***	73.4

Abbreviations: CG, control group; IG, intervention group; SMD, standardized mean difference.

* *p* < 0.05.

** *p* < 0.01.

*** *p* < 0.001.

A moderate‐high level of heterogeneity (*I* ^2^ > 50%) was found for several quality of life measures, therefore subgroup analyses were performed to verify whether type of exercise, gender, age and BMI categories explained part of that heterogeneity (Table S3). Subgroup analysis showed that combined aerobic + resistance exercise programs were more effective to improve physical subcomponents (Physical functioning: 0.77, 95% CI 0.53–1.00; Role‐physical: 0.73, 95% CI 0.41–1.05; Bodily pain: 0.51, 95% CI 0.28–0.74; General health: 0.44, 95% CI 0.14–0.73), while aerobic‐only and resistance‐only exercise programs showed non‐significant effects. A similar trend was observed for the overall physical component. On the other hand, subgroup analysis showed that only some mental subcomponents responded differently to distinct types of exercise. Specifically, social functioning and role‐emotional subcomponents were only improved with combined aerobic + resistance exercise programs (0.67, 95% CI 0.28–1.06 and 0.47, 95% CI 0.24–0.70, respectively).

Subgroup analysis by gender generally showed non‐significant differences in changes in quality of life measures, except for vitality (*Q* = 5.23, p = 0.022) and mental health (*Q* = 3.99, p = 0.046), for which exercise effects were significant only for women (1.07, 95% CI 0.32–1.82 and 0.44, 95% CI 0.17–0.72, respectively).

Subgroup analyses by age categories showed non‐significant differences in general, except for the overall mental component (*Q* = 8.87, p = 0.012) and the role‐emotional subcomponent (*Q* = 9.47, p = 0.009). The overall mental component was significantly improved by exercise in adults 40–65 years (0.34, 95% CI 0.02–0.77) and above 65 years (1.41, 95% CI 0.21–2.61). The role‐emotional subcomponent improved significantly in younger (<40 years) and older (>65 years) following exercise (0.59, 95% CI 0.17–1.01 and 0.34, 95% CI 0.06–0.62, respectively).

Subgroup analysis by BMI categories showed non‐significant differences, except for the general health subcomponent (*Q* = 10.2, p = 0.017), which was significantly improved only in class II (0.48, 95% CI 0.02–0.95) and III (0.74, 95% CI 0.26–1.22) obesity participants.

Sensitivity analyses removing outliers (when present) and poor quality studies did not lead to substantial changes in results.

There was no evidence of publication bias, except for the overall physical component and vitality. Although the heterogeneity was moderate to high, applying the trim‐and‐fill method did not substantially change the effect size in both cases.

### 3.4 Exercise‐induced effects on depression

Eight RCT studies reported results on depression using diverse instruments. The Beck Depression Inventory and the Profile of Mood Scale were used twice, and the Geriatric Depression Scale, the Cardiac Depression Scale, the Brief Symptom Inventory‐18, and the Hospital Anxiety and Depression Scale were used once. Only two of these studies (25%) found exercise‐induced improvements in depression scores.^50^, ^51^

A total of 478 participants (273 from intervention groups and 205 from controls), derived from five studies (eight arms) were included in the meta‐analysis. A non‐significant pooled effect of exercise was found (−0.13, 95% CI −0.53 to 0.26), with a moderate‐high level of heterogeneity (*I* ^2^ = 73.4). Subgroup analyses showed no significant differences across types of exercise, gender, age categories or BMI categories (Table S3). Sensitivity analysis removing poor quality studies inverted the pooled effect size direction but kept it non‐significant. No evidence of publication bias was found.

### 3.5 Exercise‐induced effects on body image

Four RCT studies measured body image using different instruments. Two studies used the Physical Self‐Perceptions Profile to assess attractiveness and physical self‐worth, one study used the Body Esteem Scale, and one study measured body satisfaction. No measures of the body image investment component (i.e., cognitive‐behavioral salience of appearance to one's Self) were used. Body satisfaction significantly increased in a combined aerobic + resistance exercise group compared to a no‐intervention control.^52^ Body esteem was not significantly altered by the addition of resistance exercise to caloric restriction compared to a diet‐only group.^53^ Body attractiveness was not significantly changed in two studies, one involving a multicomponent program with aerobic, resistance and flexibility exercise^48^ and another aerobic + resistance exercise.^54^ Physical self‐worth improved only in the study involving combined aerobic + resistance exercise.^54^

### 3.6 Exercise‐induced effects on other general psychological measures

Three RCT studies measured anxiety using three different instruments, the State–Trait Anxiety Inventory, the Brief Symptom Inventory‐18, and one with the Hospital Anxiety and Depression Scale. Positive exercise effects, from a walking program, were reported in one of these studies.^46^ The other two did not find significant effects on anxiety either in aerobic‐only or resistance‐only exercise programs.^55^, ^56^

Two RCT studies measured perceived stress using the Perceived Stress Scale.^53^, ^56^ No significant effects of either aerobic‐only or resistance‐only exercise were reported.

Two RCT studies measured self‐esteem using the Rosenberg Self‐Esteem Scale. One reported significant improvement in self‐esteem in a combined aerobic + resistance exercise program compared to a control,^54^ while the other using resistance training did not find significant changes.^53^

Two RCT studies measured life satisfaction through the Life Satisfaction Questionnaire and the Satisfaction with Life Scale. One study reported improvements in life satisfaction following a multicomponent program—involving aerobic, resistance, and flexibility exercises—compared to controls^48^ and the other did not report significant changes in three distinct exercise groups (body pump, personal training and unsupervised exercise) compared to a non‐intervention control.^57^

Subjective pain was measured in four studies (three RCT and one single‐group intervention study). Two studies used the Brief Pain Inventory and two used the Western Ontario and McMaster Universities Osteoarthritis Index (WOMAC). Aquatic exercise led to improvements in pain compared to a control group,^45^ while a combined aerobic + resistance program did not change pain scores compared to controls.^40^ The addition of combined aerobic + resistance exercise to diet restriction resulted in significant reductions in pain compared to a diet‐only group.^41^ The single‐group intervention study found significant improvements in pain scores throughout a rebound exercise intervention.^58^

### 3.7 Exercise‐induced effects on context‐specific psychological measures

Regulations/motivations for exercise were measured in two RCT studies using the Behavioural Regulation for Exercise Questionnaire. Both reported increased in autonomous motivations for exercise (i.e., intrinsic, integrated and identified regulations) in different exercise groups—combined aerobic + resistance, resistance‐only, unsupervised—compared to non‐intervention controls.^57^, ^59^ Decreases in external regulation for exercise were also found in one of these studies.^59^ Interest/enjoyment for physical activity was reported to increase in aerobic‐only and resistance‐only exercise programs (vs. controls), while skill development motives for physical activity increased only in the resistance group.^55^

Exercise self‐efficacy was measured in three occasions using the Exercise Self‐Efficacy Scale, the SERPA scale, and one did not report the instrument. All studies reported significant increases in exercise self‐efficacy in combined, aerobic‐only and resistance‐only exercise programs.^55^, ^60^, ^61^

Other specific self‐efficacy measures were used once or twice. Positive exercise effects were reported for walking self‐efficacy,^40^, ^62^ gait self‐efficacy,^62^ balance activities self‐efficacy,^62^ and schedule self‐efficacy.^36^ No effects were found for weight self‐efficacy,^53^ task self‐efficacy,^36^ barrier self‐efficacy,^36^ and stair‐climbing self‐efficacy.^40^ Occupational job stress decreased in the exercise group compared to controls, and job satisfaction did not change significantly.^63^

## 4 DISCUSSION/CONCLUSION

To inform and improve the design of future exercise interventions to foster psychological functioning and wellbeing in adults with overweight and obesity, so crucial to successful weight management efforts, the current systematic review and meta‐analysis aimed to identify and summarize the effects of supervised or semi‐supervised exercise on multiple psychological outcomes in adults with overweight or obesity and to explore these effects across different types of exercise, gender, age and BMI categories.

This systematic review suggests that exercise can effectively improve quality of life, but is not able to successfully reduce depression‐related outcomes. It also shows that most psychological outcomes (i.e., body image, anxiety, perceived stress, life satisfaction, subjective pain, and some context‐specific measures) are poorly studied and evidence either conflicting findings or null exercise effects to allow solid conclusions to be drawn. Evidence of positive exercise‐induced changes in exercise self‐efficacy and exercise autonomous motivations was found and, although limited, it was relatively consistent.

This review corroborates conclusions from prior reviews,^18^, ^19^, ^20^ specifically concerning the scarcity of studies per most psychological outcomes, the moderate‐to‐high level of heterogeneity observed for the only two psychological outcomes that allowed the use of meta‐analyses (i.e., quality of life and depression), and the large variability in intervention characteristics (length, exercise component(s), session duration and frequency); all aspects that make our efforts to interpret and draw conclusions tentative at best.

In line with ten Hoor et al.'s^20^ review on psychological effects of strength exercise in adults with overweight and obesity, as well as with prior reviews not specifically targeting this population,^17^, ^64^, ^65^ the current review identified positive changes in quality of life. However, it contradicts the findings from two other reviews, which did not find significant changes in quality of life. Possible explanations for these incongruous results might derive from the fact that one of these reviews only included adults with obesity^18^ and the other only included postmenopausal women and did not isolate exercise effects, also including interventions with behavioral components.^19^

The present review further extended prior reviews by providing an insight on the effects of exercise on distinct quality of life subcomponents. Concretely, this review showed that exercise interventions might more effectively change physical quality of life, with large effect sizes being observed, but also lead to small magnitude improvements in some subcomponents of mental quality of life, namely in vitality and mental health. Also, to explore the moderate‐high heterogeneity observed, subgroup analyses were performed. Although some subgroups included very few studies, some of these results could be suggestive of some interesting trends and thus informative for future research. We found a trend towards more favorable improvements in physical and mental quality of life in exercise programs combining aerobic and resistance training. Exercise could also have a larger effect on vitality and mental health in women than in men, and result in greater improvements in the mental component of quality of life in mid‐aged/older adults compared to younger ones.

In accordance with previous reviews,^18^, ^19^, ^20^ exercise alone does not seem to be effective in reducing depression, and this appears to be relatively consistent across types of exercise, gender, age or BMI categories. These results contrast with the literature for the general population, supporting an inverse linear relationship between physical activity participation and depression levels,^66^ but also with the results from previous studies with similar populations, such as the combination of type 2 diabetes or stroke‐recovery with excess weight.^67^, ^68^ One possible explanation for these contradicting findings might be related to a floor effect, given that participants from the included studies were not clinically diagnosed with depression (as this was an exclusion criterion for this review), and thus showed relatively low levels of depression and anxiety at baseline, not leaving much margin for improvement. It is also possible that specific types of exercise or doses are required to ameliorate certain psychological outcomes. For instance, prior research has shown that yoga and stretching exercises might have a positive impact on trait anxiety and depression,^69^ and that a dose–response effect of exercise on depression and anxiety might exist.^70^ In a recent systematic review exploring the acute effects of exercise on psychological responses, it was observed that exercise could even increase the distress felt by individuals with overweight/obesity, especially if they were not recently active.^21^ The authors proposed that feelings prior (or related) to exercise could influence the extent to which they enjoyed exercise and perceived it as less fatiguing.^21^ And, effectively, it has been previously suggested that exercise‐induced effects on psychological wellbeing might depend on an individual's perceived autonomy, competence, and self‐efficacy.^71^, ^72^ Exercise is likely to be more effective if the individual's preference for exercise modality, mode or intensity is considered, as suggested in previous studies.^73^, ^74^, ^75^

Interestingly, and in coherence with the abovementioned idea, this review showed that exercise effectively improved the individual's perceived self‐efficacy and autonomous motivation (i.e., volitional choice and congruence with one's core values) for exercise. Therefore, this is encouraging, given that these effects might indirectly influence one's psychological wellbeing and functioning.^76^

The sparse number of studies exploring exercise effects on body image, anxiety, other general psychological measures (e.g., perceived stress, life satisfaction) and context‐specific measures (e.g., occupational stress, job satisfaction), and the inconsistent findings observed, prevent us from inferring and drawing robust conclusions regarding these psychological outcomes. There is a need for more studies specifically focused at this population using standardized methodologies and measures of psychological outcomes to allow comparability of results and more robust meta‐analyses. Also, a greater systematization of the characteristics of the exercise programs is required, in terms of length, session duration, components, and intensities. In fact, the low number of studies and lack of consistency between the existing ones is a major setback in the process of providing clearer conclusions and overarching guidelines for this specific population. Also, the fact that more than half of the studies were rated as having fair or poor quality might have had an impact on our results, which may reflect the lack of quality rather than an absence of exercise effects observed in some studies for some psychological outcomes. Future research should consider these limitations.

Furthermore, the psychological effects of exercise in individuals with excess weight or obesity may take longer to be perceived or may require additional complementary psychological therapies (clinical therapy, emotional regulation intervention, mindfulness), especially when comorbidity exists (anxiety, depression, etc.). Future studies may explore this relationship and test whether complementary approaches (exercise plus psychological support) promote the manifestation of the psychological benefits of exercise observed in healthy weight adults.^66^, ^77^ Other approaches and analyses may also be followed in future studies, for instance, the influence of individual versus group training exercise programs remains unknown.

In conclusion, in spite of the present lack of sufficient and consistent evidence on the favorable effects of exercise for most psychological outcomes in adults with overweight and obesity, there are some promising results regarding quality of life and some context‐specific outcome measures, namely exercise self‐efficacy and autonomous motivations. Also, no detrimental effects of exercise on any psychological outcome were evident, which suggests that exercise should still be recommended in obesity management, even more so if we consider that it also helps regulating eating behavior in a healthier way, indirectly through its positive effects on several psychological markers like body image,^25^ self‐regulation skills,^23^ or autonomous motivations.^78^ Still, more robust studies are needed to improve evidence‐based knowledge and allow the definition of comprehensive guidelines for adults with overweight and/or obesity.

## CONFLICT OF INTEREST

The authors have no conflict of interest to declare.

## AUTHOR CONTRIBUTIONS

EC and JE performed the literature search, study selection, data extraction, and quality assessment. All authors participated in the interpretation of data. EC and JE drafted the manuscript, and authors critically revised the manuscript.

## Supporting information

**Table S1.** Web of Science Search Strategy
**Table S2.** Quality of included original studies.
**Table S3.** Subgroup analyses results for exercise effects on quality of life and depression.
