# Supplementary material for: Effect of exercise training on psychological outcomes in adults with overweight or obesity: A systematic review and meta‐analysis
Source: Obes Rev. 2021 May 6;22(Suppl 4):e13261. doi: 10.1111/obr.13261 (PMC8365728; doi:10.1111/obr.13261)
Supplement: Supplementary file 1 — Table S1. Web of Science Search Strategy Table S2. Quality of included original studies. Table S3. Subgroup analyses results for exercise effects on quality of life and depression. [file OBR-22-e13261-s001.pdf]

Effect of Exercise Training on Psychological Outcomes in Adults with Overweight and Obesity: A Systematic Review and Meta-Analysis

Authors: Eliana V. Carraça[1], Jorge Encantado[2], Francesca Battista[3], Kristine Beaulieu[4], John E. Blundell[4], Luca Busetto[5,6], Marleen van Baak[7], Dror Dicker[5,8], Andrea Ermolao[3], Nathalie Farpour-Lambert[5,9], Adryan Pramono[7], Euan Woodward[5], Alice Bellicha[10,11], Jean-Michel Oppert[12]*

[1] CIDEFES, Universidade Lusófona de Humanidades e Tecnologias, Faculdade de Educação Física e Desporto. Lisboa, Portugal.

[2] APPsyCI – Applied Psychology Research Center Capabilities & Inclusion; ISPA - University Institute.

[3] Sport and Exercise Medicine Division, Department of Medicine, University of Padova, Padova, Italy.

[4] Appetite Control and Energy Balance Group (ACEB), School of Psychology, Faculty of Medicine and Health, University of Leeds, Leeds, LS2 9JT, UK

[5] European Association for the Study of obesity (EASO), Obesity Management Task Force (OMTF).

[6] Department of Medicine, University of Padova, Padova, Italy.

[7] NUTRIM School of Nutrition and Translational Research in Metabolism, Department of Human Biology, Maastricht University Medical Centre+, Maastricht, The Netherlands

[8] Department of Internal Medicine D, Hasharon Hospital, Rabin Medical Center, Sackler School of Medicine, Tel Aviv University, Tel Aviv, Israel

[9] Obesity Prevention and Care Program Contrepoids; Service of Endocrinology, Diabetology, Nutrition and Patient Education, Department of Internal Medicine, University Hospitals of Geneva and University of Geneva, Switzerland.

[10] Sorbonne University, INSERM, Nutrition and obesities; systemic approaches (NutriOmics), Paris, France.

[11] University Paris-Est Créteil, UFR SESS-STAPS, Créteil, France

[12] Assistance Publique-Hôpitaux de Paris (AP-HP), Pitié-Salpêtrière hospital, Department of Nutrition, Institute of Cardiometabolism and Nutrition; Sorbonne University, France.

Table S1. Web of Science Search Strategy

# 8	393	#5 AND #4 AND #3 AND #2 AND #1<br>**Refined by: DOCUMENT TYPES:** (ARTICLE OR EARLY ACCESS) AND **LANGUAGES:** (ENGLISH)<br>*Indexes=SCI-EXPANDED, SSCI, A&HCI, CPCI-S, CPCI-SSH, ESCI, CCR-EXPANDED, IC Timespan=All years*	
# 7	394	#5 AND #4 AND #3 AND #2 AND #1<br>**Refined by: DOCUMENT TYPES:** (ARTICLE OR EARLY ACCESS)<br>*Indexes=SCI-EXPANDED, SSCI, A&HCI, CPCI-S, CPCI-SSH, ESCI, CCR-EXPANDED, IC Timespan=All years*	
# 6	479	#5 AND #4 AND #3 AND #2 AND #1<br>*Indexes=SCI-EXPANDED, SSCI, A&HCI, CPCI-S, CPCI-SSH, ESCI, CCR-EXPANDED, IC Timespan=All years*	Edit
# 5	2,040,640	ALL=(body image OR body appreciation OR body satisfaction OR body dissatisfaction OR self-esteem OR anxiety OR depression OR motivation OR self-regulation OR quality of life OR vitality OR perceived stress OR mental health OR psychological flexibility OR self-efficacy OR life satisfaction)<br>*Indexes=SCI-EXPANDED, SSCI, A&HCI, CPCI-S, CPCI-SSH, ESCI, CCR-EXPANDED, IC Timespan=All years*	Edit
# 4	1,035,138	ALL=(clinical trial OR randomised controlled trial OR randomized controlled trial)<br>*Indexes=SCI-EXPANDED, SSCI, A&HCI, CPCI-S, CPCI-SSH, ESCI, CCR-EXPANDED, IC Timespan=All years*	Edit
# 3	170,832	ALL=(program AND (physical activity OR exercise OR aerobic exercise OR resistance exercise OR strength exercise))<br>*Indexes=SCI-EXPANDED, SSCI, A&HCI, CPCI-S, CPCI-SSH, ESCI, CCR-EXPANDED, IC Timespan=All years*	Edit
# 2	106,332	ALL=(intervention AND (physical activity OR exercise OR aerobic exercise OR resistance exercise OR strength exercise))<br>*Indexes=SCI-EXPANDED, SSCI, A&HCI, CPCI-S, CPCI-SSH, ESCI, CCR-EXPANDED, IC Timespan=All years*	Edit
# 1	70,806	ALL=((obesity OR overweight OR obese) AND adults)<br>*Indexes=SCI-EXPANDED, SSCI, A&HCI, CPCI-S, CPCI-SSH, ESCI, CCR-EXPANDED, IC Timespan=All years*	Edit

Table S3. Quality of included original studies.

References	Criteria for controlled trials														Total "Yes"	Total "No"	Total "other"	Quality rating
	1	2	3	4	5	6	7	8	9	10	11	12	13	14				
Ballin 2019	Y	Y	Y	N	Y	Y	Y	Y	Y	CD	Y	N	Y	Y	11	2	1	GOOD
Batrakoulis 2019	Y	Y	Y	N	NR	Y	Y	Y	Y	Y	Y	Y	Y	NR	11	1	3	FAIR
Cheema 2015	Y	Y	Y	CD	Y	Y	Y	N	N	CD	Y	N	Y	Y	9	3	2	GOOD
Domene 2015	Y	Y	Y	NR	NR	Y	Y	Y	Y	CD	Y	Y	Y	N	10	1	3	FAIR
Fang 2018	Y	NR	CD	N	NR	Y	Y	Y	Y	Y	Y	NR	Y	Y	9	1	4	GOOD
Focht 2005	Y	CD	Y	N	Y	Y	Y	Y	N	Y	Y	NR	Y	Y	10	2	2	GOOD
Fritz 2011	Y	Y	Y	N	NR	N	Y	Y	Y	N	Y	N	Y	Y	9	4	1	GOOD
Grant 2004	Y	CD	NR	NR	NR	Y	N	Y	N	CD	Y	N	Y	N	5	4	5	POOR
Heiestad 2016	Y	Y	Y	N	Y	Y	N	Y	N	Y	Y	N	Y	N	9	5	0	POOR
Herring 2014	Y	Y	Y	N	NR	N	Y	Y	Y	CD	Y	Y	Y	N	9	3	2	FAIR
Imayama 2011a	Y	Y	Y	N	CD	Y	Y	Y	CD	Y	Y	CD	Y	Y	10	1	3	GOOD
Imayama 2011b	Y	Y	Y	N	Y	N	Y	Y	Y	CD	Y	Y	Y	Y	11	2	1	GOOD
Levinger 2007	Y	NR	NR	N	NR	Y	Y	Y	Y	CD	Y	NR	Y	CD	7	1	6	FAIR
Levinger 2011	Y	CD	CD	N	NR	N	Y	Y	Y	CD	Y	NR	Y	N	6	3	5	FAIR
Lim 2010	Y	CD	CD	N	Y	Y	Y	Y	Y	CD	Y	Y	Y	Y	10	1	3	GOOD
Martins 2011	Y	NR	NR	N	NR	Y	Y	Y	Y	CD	Y	NR	Y	Y	8	1	5	GOOD
Megakli 2016	Y	Y	Y	CD	CD	Y	N	CD	CD	CD	Y	N	Y	Y	7	2	5	FAIR
Megakli 2017	Y	Y	Y	CD	CD	Y	N	CD	CD	CD	Y	N	Y	Y	7	2	5	FAIR
Messier 2013	Y	NR	CD	N	Y	Y	Y	Y	N	Y	Y	Y	Y	N	9	2	3	FAIR
Messier 2010	Y	NR	NR	NR	NR	CD	N	Y	N	CD	Y	NR	Y	N	4	3	7	POOR
Mihalko 2019	Y	NR	CD	N	Y	Y	Y	Y	N	Y	Y	Y	Y	N	9	3	2	FAIR
Napoli 2014	Y	Y	Y	N	Y	Y	Y	Y	Y	CD	Y	Y	Y	Y	12	1	1	GOOD
Nieman 2000	Y	NR	CD	N	NR	NR	Y	CD	Y	Y	Y	NR	Y	N	6	2	6	FAIR
Nishijima 2007	Y	Y	Y	Y	Y	Y	Y	Y	Y	CD	Y	Y	Y	Y	13	0	1	GOOD
Plotnikoff 2010	Y	Y	Y	CD	NR	Y	Y	Y	N	Y	Y	Y	Y	Y	11	1	2	GOOD
Plotnikoff 2010	Y	Y	Y	CD	Y	Y	Y	Y	N	Y	Y	Y	Y	Y	12	1	1	GOOD
Rejeski 2002	Y	NR	CD	Y	Y	Y	N	Y	N	Y	Y	NR	Y	Y	9	2	3	FAIR
Sarsan 2006	Y	Y	Y	N	NR	Y	N	Y	N	Y	Y	NR	Y	N	8	4	2	POOR
Sukala 2013	Y	Y	Y	N	NR	N	N	Y	N	Y	Y	NR	Y	N	7	5	2	POOR

Svensson 2017	Y	NR	CD	N	NR	NR	N	N	N	CD	Y	N	Y	N	3	6	5	POOR
Tamin 2018	Y	Y	Y	N	NR	NR	N	N	N	N	Y	N	Y	N	5	7	2	POOR
Vancini 2017	Y	NR	CD	N	Y	NR	Y	CD	CD	CD	Y	NR	Y	N	5	2	7	FAIR
Villareal 2011	Y	Y	Y	N	Y	Y	Y	Y	Y	CD	Y	Y	Y	Y	12	1	1	GOOD
Rica 2013	N	NA	NA	N	CD	Y	Y	Y	CD	CD	Y	N	Y	Y	6	3	5	FAIR

Criteria for controlled trials: (1) Randomized study; (2) Adequate randomization method; (3) Treatment allocation concealment; (4) Blinding treatment assignment; (5) Blinding outcome assessors; (6) Similar baseline characteristics; (7) Drop-out rate <20%; (8) Differential dropout rate between groups <15%; (9) High adherence; (10) Similar background treatments; (11) Valid and reliable outcome measures; (12) Sample size justification; (13) Pre-specified outcomes/subgroups; (14) All randomized participants analysed (ITT analysis). Criteria 1, 7 and 14 were considered fatal flaws in answered 'No'.

References	Criteria for non-controlled trials														Total "Yes"	Total "No"	Total "other"	Quality rating
	1	2	3	4	5	6	7	8	9	10	11	12	-	-	Yes	No	Other	Quality
Baillot 2012	Y	Y	N	CD	N	Y	Y	NR	Y	Y	Y	NA	-	-	7	2	3	FAIR
Cugusi 2018	N	Y	N	Y	N	Y	Y	NR	N	Y	N	NA	-	-	5	5	2	POOR
Wouters 2010	Y	Y	N	CD	N	Y	Y	NR	Y	Y	N	NA	-	-	6	3	3	FAIR

Criteria for non-controlled trials: (1) Question/objective clearly stated; (2) Eligibility criteria pre-defined; (3) Representativeness; (4) Enrolment rates; (5) Sufficient sample size; (6) Intervention clearly described; (7) Valid and reliable outcome measures; (8) Outcome assessors blinded; (9) Drop-out rate <20% or intent to treat analysis; (10) Statistical analyses examined changes in outcomes; (11) Multiple time points for outcome measurement; (12) Account for individual changes. Criteria 2, 5 and 9 were considered fatal flaws in answered 'No'.

Table S3. Subgroup analyses results for exercise effects on quality of life and depression.

Study Characteristics	Type of Exercise	Gender	Age Categories	BMI Categories
Quality of Life				
Physical component	Q=7.20, p=0.066<br>AE: 0.18 [-0.14, 0.58], n=2<br>RE: 0.48 [-0.38, 1.33], n=3<br>CE: 0.81 [0.44, 1.17], n=3<br>WE: 4.64 [-4.12, 13.4], n=2	Not tested	Q=4.87, p=0.087<br>>65y: 1.89 [0.52, 3.25], n=5<br>40-65y: 0.38 [-0.02, 0.78], n=4<br><40y: 0.27 [-0.18, 0.73], n=1	Q=1.45, p=0.694<br>OW: 0.44 [-0.06, 0.94], n=4<br>Ob1: 4.49 [-4.54, 13.5], n=2<br>Ob2: 0.52 [-0.26, 1.30], n=2<br>Ob3: 0.73 [0.26, 1.21], n=2
Mental component	Q=6.17, p=0.104<br>AE: -0.10 [-0.43, 0.22], n=2<br>RE: 0.42 [0.03, 0.81], n=3<br>CE: -0.51 [-1.42, 0.40], n=5<br>WE: 3.96 [-2.72, 10.6], n=2	Q=0.16, p=0.694<br>Mix: 0.20 [-0.01, 0.41], n=9<br>Wm: 1.14 [-3.52, 5.79], n=3	Q=8.87, p=0.012<br>>65y: 1.41 [0.21, 2.61], n=5<br>40-65y: 0.34 [0.02, 0.77], n=4<br><40y: -1.30 [-2.63, 0.03], n=3	Q=4.71, p=0.194<br>OW: -0.40 [-1.11, 0.32], n=6<br>Ob1: 3.75 [-3.36, 10.8], n=2<br>Ob2: 0.41 [0.01, 0.81], n=2<br>Ob3: 0.26 [-0.21, 0.73], n=2
Physical functioning	Q=13.0, p<0.001<br>AE: 0.21 [0.01, 0.40], n=6<br>CE: 0.77 [0.53, 1.00], n=4	Q=0.57, p=0.451<br>Mix: 0.33 [-0.01, 0.67], n=8<br>Wm: 0.57 [0.05, 1.08], n=3	Q=5.68, p=0.058<br>>65y: 0.52 [-0.01, 1.07], n=2<br>40-65y: 0.22 [-0.08, 0.53], n=7<br><40y: 0.85 [0.43, 1.28], n=2	Q=4.71, p=0.194<br>OW: 0.44 [-0.19, 1.08], n=2<br>Ob1: 0.22 [-0.13, 0.58], n=6<br>Ob2: 0.84 [0.36, 1.32], n=1<br>Ob3: 0.68 [0.01, 1.35], n=2
Role-physical	Q=12.2, p<0.001<br>AE: 0.08 [-0.10, 0.26], n=5<br>CE: 0.73 [0.41, 1.05], n=4	Q=0.67, p=0.412<br>Mix: 0.37 [0.01, 0.73], n=7<br>Wm: 0.16 [-0.21, 0.52], n=3	Q=1.09, p=0.580<br>>65y: 0.58 [-0.25, 1.41], n=2<br>40-65y: 0.16 [-0.08, 0.40], n=6<br><40y: 0.32 [-0.22, 0.87], n=2	Q=2.87, p=0.412<br>OW: 0.09 [-0.32, 0.50], n=2<br>Ob1: 0.24 [-0.20, 0.69], n=5<br>Ob2: 0.51 [0.04, 0.98], n=1<br>Ob3: 0.61 [0.03, 1.19], n=2
Bodily pain	Q=8.08, p=0.004<br>AE: 0.09 [-0.09, 0.27], n=5<br>CE: 0.51 [0.28, 0.74], n=4	Q=0.08, p=0.780<br>Mix: 0.23 [0.03, 0.43], n=7<br>Wm: 0.29 [-0.07, 0.65], n=3	Q=9.47, p=0.009<br>>65y: 0.27 [-0.33, 0.87], n=2<br>40-65y: 0.13 [-0.04, 0.31], n=6<br><40y: 0.53 [0.12, 0.95], n=2	Q=5.77, p=0.123<br>OW: 0.01 [-0.40, 0.42], n=2<br>Ob1: 0.22 [-0.01, 0.44], n=5<br>Ob2: 0.62 [0.14, 1.09], n=1<br>Ob3: 0.32 [-0.16, 0.79], n=2

Study Characteristics	Type of Exercise	Gender	Age Categories	BMI Categories
General health	Q=8.52, p=0.014<br>AE: 0.02 [-0.16, 0.20], n=6<br>RE: 0.44 [0.14, 0.73], n=4<br>CE: -0.31 [-0.80, 0.19], n=3	Q=1.69, p=0.194<br>Mix: 0.21 [0.03, 0.38], n=8<br>Wm: -0.02 [-0.31, 0.27], n=6	Q=1.24, p=0.538<br>>65y: 0.15 [-0.11, 0.41], n=2<br>40-65y: 0.20 [-0.03, 0.43], n=7<br><40y: -0.09 [-0.53, 0.36], n=5	Q=10.2, p=0.017<br>OW: -0.05 [-0.48, 0.37], n=2<br>Ob1: 0.05 [-0.10, 0.21], n=9<br>Ob2: 0.48 [0.02, 0.95], n=1<br>Ob3: 0.74 [0.26, 1.22], n=2
Vitality	Q=3.41, p=0.065<br>AE: 0.22 [0.04, 0.39], n=6<br>CE: 0.81 [0.21, 1.42], n=6	Q=5.23, p=0.022<br>Mix: 0.17 [-0.00, 0.34], n=8<br>Wm: 1.07 [0.32, 1.86], n=5	Q=4.94, p=0.084<br>>65y: 0.13 [-0.13, 0.39], n=2<br>40-65y: 0.20 [0.03, 0.38], n=7<br><40y: 1.40 [0.31, 2.48], n=4	Q=5.13, p=0.163<br>OW: 1.35 [0.16, 2.54], n=4<br>Ob1: 0.15 [-0.01, 0.32], n=6<br>Ob2: 0.39 [-0.08, 0.85], n=1<br>Ob3: 0.41 [-0.09, 0.92], n=2
Social functioning	Q=7.65, p=0.006<br>AE: 0.06 [-0.12, 0.24], n=5<br>CE: 0.67 [0.28, 1.06], n=4	Q=0.02, p=0.877<br>Mix: 0.27 [-0.15, 0.69], n=7<br>Wm: 0.23 [-0.05, 0.51], n=3	Q=3.54, p=0.170<br>>65y: 0.59 [-0.41, 1.59], n=2<br>40-65y: 0.08 [-0.09, 0.26], n=6<br><40y: 0.47 [0.05, 0.88], n=2	Q=1.96, p=0.580<br>OW: 0.11 [-0.30, 0.52], n=2<br>Ob1: 0.22 [0.28, 0.72], n=5<br>Ob2: 0.52 [0.05, 0.99], n=1<br>Ob3: 0.41 [-0.07, 0.88], n=2
Role-emotional	Q=10.8, p=0.001<br>AE: -0.01 [-0.20, 0.16], n=5<br>CE: 0.47 [0.24, 0.70], n=4	Q=0.19, p=0.663<br>Mix: 0.15 [-0.07, 0.36], n=7<br>Wm: 0.28 [-0.26, 0.81], n=3	Q=9.47, p=0.009<br>>65y: 0.34 [0.06, 0.62], n=2<br>40-65y: -0.02 [-0.20, 0.16], n=6<br><40y: 0.59 [0.17, 1.01], n=2	Q=5.77, p=0.123<br>OW: 0.17 [-0.24, 0.58], n=2<br>Ob1: 0.04 [-0.23, 0.30], n=5<br>Ob2: 0.68 [0.20, 1.15], n=1<br>Ob3: 0.34 [-0.13, 0.81], n=2
Mental health	Q=0.56, p=0.454<br>AE: 0.19 [0.01, 0.38], n=5<br>CE: 0.31 [0.07, 0.54], n=4	Q=3.99, p=0.046<br>Mix: 0.11 [-0.06, 0.29], n=7<br>Wm: 0.44 [0.17, 0.72], n=3	Q=5.17, p=0.076<br>>65y: 0.15 [-0.11, 0.40], n=2<br>40-65y: 0.17 [-0.01, 0.35], n=6<br><40y: 0.68 [0.26, 1.10], n=2	Q=4.19, p=0.242<br>OW: 0.39 [-0.28, 1.06], n=2<br>Ob1: 0.15 [-0.02, 0.31], n=5<br>Ob2: 0.63 [0.16, 1.10], n=1<br>Ob3: 0.34 [-0.13, 0.81], n=2
Depression	Q=0.35, p=0.552<br>AE: -0.16 [-0.51, 0.19], n=4<br>RE: -0.61 [-2.03, 0.82], n=2	Q=2.95, p=0.086<br>Mix: 0.17 [-0.27, 0.61], n=5<br>Wm: -0.62 [-1.41, 0.16], n=3	Q=0.004, p=0.953<br>>65y: -0.15 [-0.46, 0.24], n=3<br>40-65y: -0.14 [-0.79, 0.52], n=5	Q=1.57, p=0.456<br>OW: -0.13 [-0.61, 0.35], n=2<br>Ob1: 0.14 [-0.44, 0.71], n=4<br>Ob2: -0.70 [-1.92, 0.52], n=2

Legend: AE, aerobic exercise; RE, resistance exercise; CE, combined aerobic + resistance exercise; WE, water-based exercise; Mix, mixed gender samples; Wm, women-only samples; >65y, older adults; 40-65y, mid-age adults; <40y, younger adults; OW, overweight; Ob1, Class I Obesity; Ob2, Class II Obesity; Ob3, Class III Obesity; Cochran's Q statistic and p-values indicate subgroup differences in effects.
